# Epidemiological profile of the homeless population and factors associated with illicit psychoactive substance use: cross-sectional study, Jundiaí, 2023.

**DOI:** 10.1590/S2237-96222026v35e20240124.en

**Published:** 2026-04-22

**Authors:** Gabriel Vinícius Reis de Queiroz, Tatiane Bahia do Vale Silva, Marília Jesus Batista

**Affiliations:** 1 Faculdade de Medicina de Jundiaí, Programa de Pós-Graduação em Ciências da Saúde, Jundiaí, SP, Brazil Faculdade de Medicina de Jundiaí Programa de Pós-Graduação em Ciências da Saúde Jundiaí SP Brazil; 2 Universidade do Estado do Pará, Departamento de Ciências do Movimento Humano, Belém, PA, Brazil Universidade do Estado do Pará Departamento de Ciências do Movimento Humano Belém PA Brazil; 3 Faculdade de Medicina de Jundiaí, Departamento de Saúde Coletiva, Jundiaí, SP, Brazil Faculdade de Medicina de Jundiaí Departamento de Saúde Coletiva Jundiaí SP Brazil

**Keywords:** Ill-Housed Persons, Social Vulnerability, Epidemiology, Illicit Drugs, Cross-Sectional Studies, Personas con Mala Vivienda, Vulnerabilidad Social, Epidemiología, Drogas Ilícitas, Estudios Transversales

## Abstract

**Objectives:**

To describe the sociodemographic and clinical-epidemiological characteristics of the homeless population and to assess factors associated with illicit psychoactive substance use in Jundiaí, São Paulo, Brazil, in 2023.

**Methods:**

: This study employed a cross-sectional design with a homeless population. The outcome was substance use, and the independent variables were grouped into exogenous factors, primary and intermediate health determinants, access to services, health perception, and clinical conditions. Descriptive and bivariate statistical analyses were performed. Odds ratios (OR) with 95% confidence intervals (95%CI) were calculated using multiple binary logistic regression.

**Results:**

There were 270 participants, the majority of whom were male (85.2%), with an average age of 39, self-identified as Black (72.6%), heterosexual (88.5%), with incomplete elementary education (54.8%), living on the street for five years or more (45.6%), and 80.4% had used/were using illicit psychoactive substances. The most prevalent conditions were mental distress (83.0%), sleep problems (77.4%), digestive discomfort (34.8%), and respiratory disease (31.5%). The use of illicit substances was associated with: having completed high school or higher (OR 4.16; 95%CI 1.63; 10.62); living on the streets for more than one year (OR 2.08; 95%CI 1.01; 4.28); having suffered violence (OR 2.05; 95%CI 1.04; 4.04); smoking (OR 3.46; 95%CI 1.60; 7.50); and not receiving social assistance benefits (OR 2.62; 95%CI 1.28; 5.34).

**Conclusion:**

The study found comorbidities and a high prevalence of illicit psychoactive substance use associated with socioeconomic and behavioral health determinants. These data may inform public health management in developing strategies tailored to the specific needs of this population.

## Resumen

**Objetivos:**

Describir las variables sociodemográficas y clínico-epidemiológicas de la población en situación de calle y evaluar los factores asociados al consumo de sustancias psicoactivas ilícitas en Jundiaí, São Paulo, Brasil, en 2023.

**Métodos:**

Estudio transversal con una población en situación de calle; el desenlace fue el consumo de sustancias, y las variables independientes se agruparon en exógenas, determinantes primarios e intermedios en salud, acceso a servicios, percepción de la salud y condiciones clínicas. Se utilizó análisis estadístico descriptivo y bivariado. Se calcularon las razones de probabilidad (odds ratio, OR) con intervalos de confianza del 95 % (IC95 %) mediante regresión logística binaria múltiple.

**Resultados:**

Participaron 270 personas, en su mayoría hombres (85,2 %), con una edad media de 39 años, que se declararon negros (72,6 %), heterosexuales (88,5 %), con estudios de primaria incompletos (54,8 %), en situación de calle durante cinco años o más (45,6 %) y el 80,4 % ya habían consumido o consumían sustancias psicoactivas ilícitas. Los trastornos más prevalentes fueron el sufrimiento mental (83,0 %), los problemas de sueño (77,4 %), el malestar digestivo (34,8 %) y las enfermedades respiratorias (31,5 %). El consumo de sustancias ilícitas se asoció con: haber completado la educación secundaria o superior (OR 4,16; IC95 % 1,63; 10,62); llevar más de un año en situación de calle (OR 2,08; IC95 % 1,01; 4,28); haber sufrido violencia (OR 2,05; IC95% 1,04; 4,04); tabaquismo (OR 3,46; IC95% 1,60; 7,50); y no recibir prestaciones sociales (OR 2,62; IC95% 1,28; 5,34).

**Conclusión:**

Se observaron comorbilidades y una alta prevalencia del consumo de sustancias psicoactivas ilícitas asociadas a determinantes socioeconómicos y conductuales en materia de salud. Estos datos orientan la gestión pública de la salud en la elaboración de estrategias para las necesidades específicas de esta población.

**Ethical aspects**

This research respected ethical principles, having obtained the following approval data:

Research ethics committee: Faculdade de Medicina de Jundiaí

Opinion number: 4915622

Approval date: 18/8/2021

Certificate of submission for ethical appraisal: 48459121.4.0000.5412

Informed consent record: Obtained from all participants prior to data collection.

## Introduction

The existence of homeless individuals is a global phenomenon. It represents a stark manifestation of inequality and social exclusion resulting from the overvaluation of capital to the detriment of human rights ^1^. This population group experiences weakened or nonexistent family ties, extreme poverty, and a lack of conventional housing, often using public spaces and/or shelters as their places of residence ^2^. According to the 2021 Census conducted by the São Paulo City Government, following the onset of the COVID-19 pandemic, a 31.0% increase was observed in the homeless population in the city of São Paulo, rising from 24,344 individuals in 2019 to 31,884 in 2021. In addition, it is estimated that 281,472 people live in this condition across Brazil, highlighting a significant challenge for the country’s social policies ^3^^,^^4^.

The issue of vulnerability, along with the cultural specificities and lifestyles of the homeless population, directly impacts the social determinants of health ^5^^,^^6^. People experiencing homelessness are constantly exposed to precarious conditions in terms of health, hygiene, housing, and food; excessive exposure to heat and cold; violence; and/or exhausting physical efforts for survival, which may result in the emergence or worsening of health problems ^6^. Literature indicates that the most prevalent health conditions among this population include tuberculosis, pneumonia, syphilis, human immunodeficiency virus, genital herpes, dental pain, hypertension, mental distress, intestinal parasitosis, and the abusive use of psychoactive substances ^7^^,^^8^^,^^9^.

The reasons that lead individuals to live on the streets vary, with domestic violence, personal tragedies, family conflicts, and psychoactive substance use being among the most frequent causes ^10^. The use of these substances is everyday and serves as a means of coping with a harsh reality ^11^. To understand this use, it is important to view these individuals as protagonists of their own stories, taking into account their life experiences and social contexts, as well as their need for support networks and cultural influences ^10^^,^^11^.

This study is relevant as it addresses the health conditions of the homeless population, promoting visibility for a socially vulnerable group. Such an initiative contributes to reducing health inequalities and combating persistent stigma and prejudice. It is therefore essential to understand the characteristics of this population in order to develop public policies that are appropriate and reflective of their specific needs. This is crucial for improving health care strategies based on the lived reality of this population and an understanding of their cultural dynamics, particularly with regard to psychoactive substance use.

Accordingly, the objective of this study is to describe the sociodemographic and clinical-epidemiological variables of the homeless population and to assess factors associated with illicit psychoactive substance use in Jundiaí, São Paulo, Brazil, in 2023.

## Methods

### Design

An analytical cross-sectional epidemiological study was reported in accordance with the Strengthening the Reporting of Observational Studies in Epidemiology (STROBE) guidelines for observational studies.

### Setting

The study population consisted of homeless people in Jundiaí, São Paulo, Brazil ^12^. This city is located 57 km from the state capital, São Paulo, and according to the 2022 census conducted by the Brazilian Institute of Geography and Statistics, the estimated population was 443,221 inhabitants, with a human development index of 0.822 ^13^. 

### Data collection

Data collection was conducted from August 2022 to September 2023, with the assistance of the Consultório na Rua (Street Clinic, in literal translation) team. This service, created in Jundiaí in April 2014, assists socially vulnerable people, mainly those who use psychoactive substances and are disconnected from healthcare networks. In Jundiaí, the Consultório na Rua team has a coordinator, a psychologist, two nurses, two harm reduction workers, a family doctor, and a psychiatrist.

The Consultório na Rua team works in areas with a high concentration of homeless people, focusing on the outskirts where the use of psychoactive substances is everyday. During meetings with researchers, the importance of integrating the various activities was noted. Contact with the population revealed initial resistance on their part. Nevertheless, with explanations about the research and the frequency of visits, it was possible to establish a dialogue that allowed the study to be conducted.

### Participants

The present study included homeless individuals in Jundiaí, of both sexes, aged 18 years or older. Individuals in transit (those passing through the municipality on their way to another location), those under the influence of psychoactive substances (alcohol and/or drugs) at the time of data collection, and individuals with oral speech impairment were excluded.

### Variables

A conceptual theoretical model was adapted for the selection of variables ^14^. This model, developed by Andersen and Davidson, aims to identify the essential variables for a systematic analysis of the factors that influence health diversity ^15^. According to this model, external environmental conditions, the healthcare structure, and the individual characteristics of the population have a considerable influence on health-related behaviors ^15^. The variables in this study are grouped into the following blocks, starting from the most distal to the most proximal (Figure 1).

**Figure 1.**
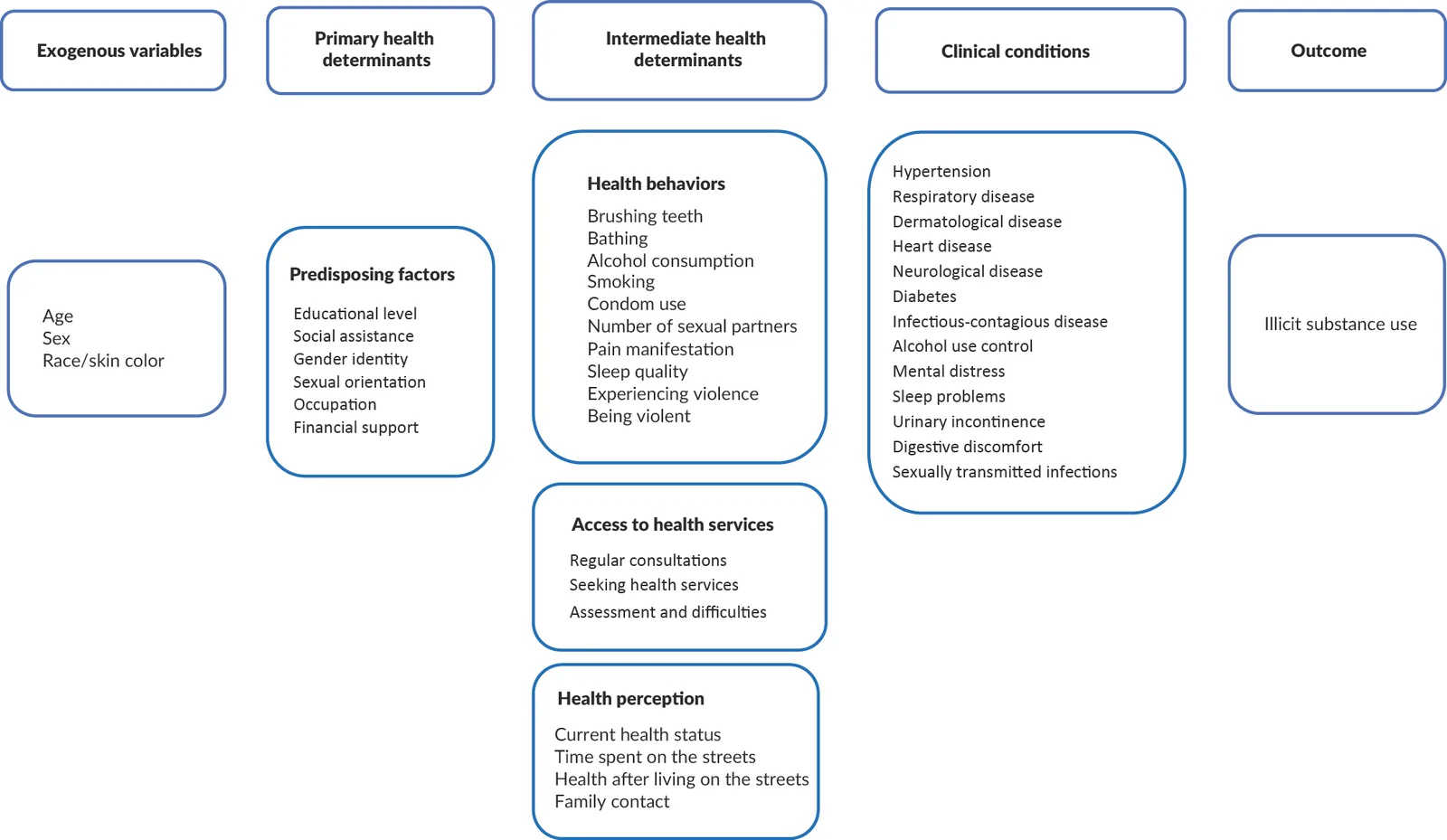
A conceptual theoretical model for the selection of variables

Exogenous: age (continuous), sex (male; female), and race/skin color (White; Black; Brown; other [Indigenous; Asian]).

Primary health determinants: gender identity (cis; trans), sexual orientation (heterosexual; homosexual; bisexual; pansexual), education (high school graduate or higher; elementary school graduate; incomplete elementary school), having a profession (yes; no), financial livelihood (commerce/handicrafts; construction; car guard; recyclable material collection; truck loader; cleaning; gardening; sex worker; other) and receiving any social assistance benefits (yes; no).

Intermediate health determinants: brushing teeth (yes; no), bathing (yes; no), consuming alcoholic beverages (yes; no), smoking (yes; no; former smoker), using condoms (yes; no), number of sexual partners (none; one or more), pain at the time of the interview (yes; no), sleep quality (very good/good; fair; poor/very poor), whether they have suffered violence (yes; no) and whether they have been violent (yes; no).

Access to health services: consultation with health professionals (rarely/never; sometimes; frequently/always), whether they seek health services when ill (yes; no), last time they saw a doctor and/or dentist (less than six months ago; six months to one year ago; one to two years ago; three years ago; more than three years ago; unknow; never sought), assessment of health services already used (very good/good; fair; poor/very poor), difficulties in accessing health services (treatment by staff; difficulty in getting an appointment; unfamiliarity with services; lack of documents; no difficulties); Possible forms of treatment by staff included racism, mistreatment, violence, and disorganization.

Perception of health: classification of current health status (very good/good; fair; poor/very poor), time spent homeless (up to one year; more than one year), health after becoming homeless (improved/improved a lot; remained the same; worsened/worsened a lot), and contact with family members (up to one week; up to one year; lost contact/more than five years).

Clinical conditions: high blood pressure (yes; no), respiratory disease (yes; no), skin disease (yes; no), heart disease (yes; no), neurological disease (yes; no), diabetes (yes; no), infectious disease (yes; no), difficulty controlling alcohol use (yes; no), mental distress (yes; no), sleep problems (yes; no), urinary incontinence (yes; no), digestive discomfort (yes; no), and sexually transmitted infections (yes; no), both self-reported. 

The dependent variable (outcome) of this study was illicit substance use, with “yes” (currently uses and has used) for illicit substance use and “no” (does not use and has never used) when the participant reported not using these substances. In order to describe the characteristics of this population, questions were asked about the types of illicit substances used, such as crack, marijuana, cocaine, ecstasy, and inhaled drugs.

### Data sources and measurement

A questionnaire based on the Situation and Vulnerability Instrument was explicitly developed for the homeless population and adapted for the present study to collect information on sociodemographic aspects and living and health conditions ^16^. The questionnaire consisted of a total of 58 questions, including both open-ended and closed-ended items. Initially, the questionnaire was validated by the Consultório na Rua team, and, subsequently, a pilot study was conducted in the territory with ten individuals. 

The interviews were conducted by the lead researcher and members of the Consultório na Rua team, all of whom were previously trained. Participants were first approached in different areas of the city, informed about the study, and invited to participate. Upon confirmation of their participation, the questionnaire was administered to them. Whether on the street or during care provided at service units, data collection was conducted in more private settings whenever possible.

### Study size

Given the transient nature of this population group, convenience sampling was employed, enabling the inclusion of homeless individuals from various areas of the city while the Consultório na Rua team provided services.

Sampling was carried out using the appropriate formula for finite populations. For the sample size calculation, a study on the health of the homeless population in Belo Horizonte, Minas Gerais, was used as a reference, which found that 47.35% of respondents rated their health as poor ^17^. A significance level of 5% and a 10% margin of error were adopted. The final sample size was estimated at 216 participants. To account for potential losses, 20.0% was added to the sample, resulting in a total of 270 participants.

### Statistical methods

The data collected were processed using the Statistical Package for the Social Sciences (SPSS), version 20.0. Descriptive analyses were first performed to obtain frequency distributions and the mean age. Bivariate analyses were then conducted between the outcome-illicit substance use (yes/previous use vs. no/never used)-and the independent variables using the Chi-square test. 

Univariate binary logistic regression analysis was performed. Variables with a p-value<0.20 were included in multiple binary logistic regression models using the step forward method. Multicollinearity was tested. The exponential β in this study was presented as the crude odds ratio (OR) for the univariate analysis and the adjusted OR following logistic regression. A 95% confidence interval (95%CI) was also calculated. A significance level of 5% was adopted.

## Results

Among the 270 homeless individuals included in this study, with a mean age of 39 years, 85.2% self-identified as male, 72.6% as Black or Brown, 88.5% as heterosexual, and 54.8% had not completed elementary school. A total of 28.5% reported collecting recyclable materials as their primary source of income, 47.8% did not receive any social assistance benefits, and 45.6% had been living on the streets for more than five years. The most prevalent reasons for homelessness were the use of psychoactive substances (27.4%) and personal tragedies (21.1%). It was found that 80.4% of participants were currently using or had previously used some illicit substance; 48.1% reported using crack, 41.5% marijuana, and 33.0% cocaine (Table 1).

Regarding clinical conditions, 24.4% of participants reported having hypertension, 31.5% had a respiratory disease, 33.0% reported difficulty controlling alcohol use, 83.0% experienced mental distress, 77.4% had sleep problems, 22.6% reported urinary incontinence, and 34.8% digestive discomfort. Concerning health service use, 58.5% rated the services as good, and 67,0% reported no difficulty in accessing them. However, 22.6% mentioned experiencing barriers related to how they were treated by health workers, including racism, mistreatment, violence, and/or disorganization (Table 2).

**Table 1:** Frequency (n) and percentage (%) of sociodemographic characteristics and health behaviors of the homeless population. Jundiaí, 2023 (n=270)

Variables			n (%)
**Sociodemographic characteristics**	Race/skin color	White	69 (25.6)
		Black	56 (20.7)
		Brown	140 (51.9)
		Other (Indigenous, Asian)	5 (1.8)
	Sex	Male	230 (85.2)
		Female	40 (14.8)
	Gender identity^a^	Cis	256 (94.8)
		Trans	13 (4.8)
	Sexual orientation	Heterosexual	239 (88.5)
		Homosexual	17 (6.3)
		Bisexual	13 (4.8)
		Pansexual	1 (0.4)
	Education level	Incomplete elementary school	148 (54.8)
		Complete elementary school	52 (19.3)
		Complete high school or higher	70 (25.9)
	Having a profession	Yes	255 (94.4)
		No	15 (5.6)
	Receiving any social assistance benefits	Yes	141 (52.2)
		No	129 (47.8)
	Financial livelihood	Commerce/handicrafts	27 (10.0)
		Civil construction	23 (8.5)
		Car guard	3 (1.1)
		Recyclable material collection	77 (28.5)
		Truck loader	3 (1.1)
		Cleaning	4 (1.5)
		Gardening	4 (1.5)
		Sex worker	11 (4.1)
		Other	56 (20.7)
		Do not work	62 (23.0)
	Time spent homeless	Up to one year	78 (28.9)
		From one to four years and 11 months	68 (25.2)
		More than five years	123 (45.6)
		Unknown	1 (0.3)
**Health behaviors**	Classification of current health status^c^	Very good	6 (2.2)
		Good	46 (17.0)
		Fair	82 (30.4)
		Poor	63 (23.3)
		Very poor	73 (27.0)
	Feeling pain at the time of the interview	Yes	140 (51.9)
		No	130 (48.1)
	Health condition after becoming homeless	Improved a lot	3 (1.1)
		Improved	8 (3.0)
		Remained the same	50 (18.5)
		Worsened	57 (21.1)
		Worsened a lot	152 (56.3)
**Health behaviors**	Reason for being homeless^c^	Job-seeking	19 (7.0)
		Domestic violence	47 (17.4)
		Abandonment by family	34 (12.6)
		Threats/conflicts	2 (0.7)
		Release from prison	8 (3.0)
		Loss of everything	16 (5.9)
		Personal tragedies	57 (21.1)
		Discrimination based on sexual orientation	4 (1.5)
		Use of psychoactive substances	74 (27.4)
		Other	7 (2.6)
		Unknown	2 (0.7)
	Brushing teeth	Yes	225 (83.3)
		No	45 (16.7)
	Bathing	Yes	259 (95.9)
		No	11 (4.1)
	Have suffered violence	Yes	160 (59.3)
		No	110 (40.7)
	Sleep quality	Very good	5 (1.9)
		Good	38 (14.1)
		Fair	27 (10.0)
		Poor	25 (9.3)
		Very poor	175 (64.8)
	Consuming alcoholic beverages^c^	Yes	183 (67.8)
		No	86 (31.9)
		Unknown	1 (0.4)
	Smoker	Yes	211 (78.1)
		No	47 (17.4)
		Former smoker	12 (4.4)
	Illicit substance use	Yes	179 (66.3)
		No	53 (19.6)
		Previous use	38 (14.1)
	Use of crack	Yes	130 (48.1)
		No	102 (37.8)
		Not applicable^b^	38 (14.1)
	Use of marijuana	Yes	112 (41.5)
		No	120 (44.4)
		Not applicable^b^	38 (14.1)
	Use of cocaine^c^	Yes	89 (33.0)
		No	143 (53.0)
		Not applicable^b^	38 (14.1)
	Use of ecstasy^c^	Yes	15 (5.6)
		No	217 (80.4)
		Not applicable^b^	38 (14.1)
	Use of inhaled drugs^c^	Yes	15 (5.6)
		No	217 (80.4)
		Not applicable^b^	38 (14.1)
	Condom use	Yes	155 (57.4)
		No	112 (41.5)
		Unknown	3 (1.1)

a Variable that did not total 100.0% due to missing responses (missing data); ^b^“Not applicable” refers to respondents who reported having used any illicit substance; ^c^Percentages were rounded to one decimal place. The sum may not total exactly 100.0%.

**Table 2.** Frequency (n) and percentage (%) of clinical characteristics and use of health services by the homeless population. Jundiaí, 2023 (n=270)

Variables			n (%)
Clinical conditions	Arterial hypertension^a^	Yes	66 (24.4)
		No	181 (67.0)
		Unknown	23 (8.5)
	Heart disease	Yes	17 (6.3)
		No	241 (89.3)
		Unknown	12 (4.4)
	Neurological disease	Yes	21 (7.8)
		No	237 (87.8)
		Unknown	12 (4.4)
	Infectious disease	Yes	20 (7.4)
		No	239 (88.5)
		Unknown	11 (4.1)
	Respiratory disease	Yes	85 (31.5)
		No	174 (64.4)
		Unknown	11 (4.1)
	Skin disease	Yes	32 (11.9)
		No	236 (87.4)
		Unknown	2 (0.7)
	Diabetes	Yes	20 (7.4)
		No	227 (84.1)
		Unknown	23 (8.5)
	Difficulty controlling alcohol use^a^	Yes	89 (33.0)
		No	180 (66.7)
		Unknown	1 (0.4)
	Mental distress (depression, anxiety)	Yes	224 (83.0)
		No	43 (15.9)
		Unknown	3 (1.1)
	Sleep problems	Yes	209 (77.4)
		No	60 (22.2)
		Unknown	1 (0.4)
	Urinary incontinence	Yes	61 (22.6)
		No	208 (77.0)
		Unknown	1 (0.4)
	Digestive discomfort	Yes	94 (34.8)
		No	176 (65.2)
	Sexually transmitted infections (STI)	Yes	23 (8.5)
		No	212 (78.5)
		Unknown	35 (13.0)
Service use	Healthcare appointment	Never	123 (45.6)
		Rarely	57 (21.1)
		Sometimes	56 (20.7)
		Frequently	23 (8.5)
		Always	11 (4.1)
	Seeking health services when ill	Yes	157 (58.1)
		No	112 (41.5)
		Unknown	1 (0.4)
	Last time they sought a doctor and/or dentist	Less than six months ago	158 (58.5)
		Six months to one year ago	33 (12.2)
		One to two years ago	23 (8.5)
		Three years ago	6 (2.2)
		More than three years ago	42 (15.6)
		Unknown	6 (2.2)
		Never sought	2 (0.7)
	Last time they sought a doctor and/or dentist	Less than six months ago	48 (17.8)
		Six months to one year ago	27 (10.0)
		One to two years ago	33 (12.2)
		Three years	2 (0.7)
		More than three years ago	126 (46.7)
		Unknown	5 (1.9)
		Never sought	29 (10.7)
	Assessment of health services already used	Very good	47 (17.4)
		Good	158 (58.5)
		Fair	39 (14.4)
		Poor	11 (4.1)
		Very poor	15 (5.6)
	Difficulties in accessing health services	The way staff treated	61 (22.6)
		Difficulty in getting an appointment	11 (4.1)
		Unfamiliarity with services	12 (4.4)
		Lack of documents	5 (1.9)
		No difficulties)	181 (67.0)

a Percentages have been rounded to one decimal place. The sum may not total exactly 100.0%.

The bivariate analysis revealed an association between illicit substance use (outcome) and the following variables: having completed high school or higher education (OR 3.22; 95%CI 1.36; 7.62), not receiving social assistance benefits (OR 2.51; 95%CI 1.31; 4.78), being a smoker (OR 4.14; 95%CI 2.07; 8.26), having experienced violence (OR 2.23; 95%CI 1.21; 4.10), having committed violence (OR 2.56; 95%CI 1.38; 4.75), being homeless for more than one year (OR 2.02; 95%CI 1.08; 3.79), not having hypertension (OR 2.10; 95%CI 1.09; 4.03), and not having diabetes (OR 4.40; 95%CI 1.72; 11.25) (Table 3).

In the final regression model, the variables that remained significantly associated with illicit substance use were: having completed high school or higher education (OR 4.16; 95%CI 1.63; 10.62), not receiving social assistance benefits (OR 2.62; 95%CI 1.28; 5.34), having experienced violence (OR 2.05; 95%CI 1.04; 4.04), being a smoker (OR 3.46; 95%CI 1.60; 7.50), and being homeless for more than one year (OR 2.08; 95%CI 1.01; 4.28) (Table 4).

**Table 3:** Odds ratio (OR) and 95% confidence intervals (95%CI) for illicit substance use and its association with exogenous variables, primary and intermediate determinants of health, and clinical conditions among homeless people. Jundiaí, 2023 (n=270)

Variables			Illicit substance use			
			Yes	No	p-value	OR (95%IC)
			n (%)	n (%)		
Exogenous variables	Sex	Male	185 (80.4)	45 (19.6)	0.949	1.03 (0.44; 2.38)
		Female	32 (80.0)	8 (20.0)		1.00
	Race/skin color	White	55 (79.7)	14 (20.3)	0.616	1.00
		Black	47 (83.9)	9 (16.1)		1.33 (0.53; 3.35)
		Brown	112 (80.0)	28 (20.0)		1.02 (0.50; 2.09)
		Other	3 (60.0)	2 (40.0)		0.38 (0.06; 2.51)
Primary determinants	Gender identity	Cis	207 (80.9)	49 (19.1)	0.721	1.27 (0.34; 4.78)
of health		Trans	10 (76.9)	3 (23.1)		1.00
	Education level	Complete high school or higher	63 (90.0)	7 (10.0)	0.008	3.22 (1.36; 7.62)
		Complete elementary school	45 (86.5)	7 (13.5)		2.30 (0.96; 5.53)
		Incomplete elementary school	109 (73.6)	39 (26.4)		1.00
	Receiving any social assistance benefits	Yes	104 (73.8)	37 (26.2)	0.004	1.00
		No	113 (87.6)	16 (12.4)		2.51 (1.31; 4.78)
**Health behaviors**	Brushing teeth	Yes	181 (80.4)	44 (19.6)	0.945	1.00
		No	36 (80.0)	9 (20.0)		0.97 (0.44; 2.17)
	Bathing	Yes	210 (81.1)	49 (18.9)	0.154	1.00
		No	7 (63.6)	4 (36.4)		0.41 (0.12; 1.45)
	Consuming alcoholic beverages	Yes	152 (83.1)	31 (16.9)	0.147	1.00
		No	65 (75.6)	21 (24.4)		0.63 (0.34; 1.18)
	Smoker	Yes	179 (84.8)	32 (15.2)	<0.001	4.14 (2.07; 8.26)
		No	27 (57.4)	20 (42.6)		1.00
		Former smoker	11 ( 91.7)	1 ( 8.3)		8.15 (0.97; 68.38)
	Condom use	Yes	128 (82.6)	27 (17.4)	0.411	1.00
		No	88 (78.6)	24 (21.4)		0.77 (0.42; 1.43)
	Number of sexual partners	None	139 (79.0)	37 (21.0)	0.430	1.00
		One or more	78 (83.0)	16 (17.0)		1.30 (0.68; 2.48)
	Sleep quality assessment	Very good/good	31 (72.1)	12 (27.9)	0.297	1.00
		Fair	23 (85.2)	4 (14.8)		2.23 (0.64; 7.80)
		Poor/very poor	163 (81.5)	37 (18.5)		1.71 (0.80; 3.63)
	Has suffered violence	Yes	137 (85.6)	23 (14.4)	0.009	2.23 (1.21; 4.10)
		No	80 (72.7)	30 (27.3)		1.00
	Has committed violence	Yes	132 (86.8)	20 (13.2)	0.002	2.56 (1.38; 4.75)
		No	85 (72.0)	33 (28.0)		1.00
**Access to health services**	Consultation with health professionals	Rarely/never	151 (83.9)	29 (16.1)	0.063	0.74 (0.30; 1.86)
		Sometimes	39 (69.6)	17 (30.4)		0.44 (0.22; 0.82)
		Frequently/always	27 (79.4)	7 (20.6)		1.00
	Seeking health services when ill	Yes	120 (76.4)	37 (23.6)	0.059	1.00
		No	96 (85.7)	16 (14.3)		1.85 (0.97; 3.53)
**Perception of health**	Classification of current health status	Very good/good	40 (76.9)	12 (23.1)	0.776	1.00
		Fair	67 (81.7)	15 (18.3)		1.34 (0.57; 3.15)
		Poor/very poor	110 (80.9)	26 (19.1)		1.27 (0.59; 2.75)
	Time spent homeless	Up to one year	56 (71.8)	22 (28.2)	0.025	1.00
		More than one year	160 (83.8)	31 (16.2)		2.02 (1.08; 3.79)
	Health conditions after becoming homeless	Improved/improved a lot	8 (72.7)	3 (27.3)	0.782	1.00
		Remained the same	41 (82.0)	9 (18.0)		1.71 (0.38; 7.74)
		Worsened/worsened a lot	168 (80.4)	41 (19.6)		1.54 (0.39; 6.05)
	Contact with family members	Once a week	59 (81.9)	13 (18.1)	0.197	1.61 (0.77; 3.39)
		Once a year	41 (85.4)	7 (14.6)		2.08 (0.83; 5.19)
		Lost contact/more than five years	76 (73.8)	27 (26.2)		1.00
**Clinical conditions**	Arterial hypertension	Yes	46 (69.7)	20 (30.3)	0.024	1.00
		No	150 (82.9)	31 (17.1)		2.10 (1.09; 4.03)
	Respiratory disease	Yes	75 (88.2)	10 (11.8)	0.054	2.10 (0.99; 4.44)
		No	136 (78.2)	38 (21.8)		1.00
	Skin disease	Yes	26 (81.2)	6 (18.8)	0.877	1.08 (0.42; 2.77)
		No	189 (80.1)	47 (19.9)		1.00
	Heart disease	Yes	14 (82.4)	3 (17.6)	0.852	1.13 (0.31; 4.10)
		No	194 (80.5)	47 (19.5)		1.00
	Neurological disease	Yes	15 (71.4)	6 (28.6)	0.258	0.57 (0.21; 1.55)
		No	193 (81.4)	44 (18.6)		1.00
	Diabetes	Yes	10 (50.0)	10 (50.0)	0.003	1.00
		No	185 (81.5)	42 (18.5)		4.40 (1.72; 11.25)
	Infectious disease	Yes	17 (85.0)	3 (15.0)	0.774	1.35 (0.38; 4.80)
		No	193 (80.8)	46 (19.2)		1.00
	Difficulty controlling alcohol use	Yes	71 (79.8)	18 (20.2)	0.794	0.92 (0.49; 1.74)
		No	146 (81.1)	34 (18.9)		1.00
	Mental distress	Yes	180 (80.4)	44 (19.6)	0.875	0.94 (0.41; 2.16)
		No	35 (81.4)	8 (18.6)		1.00
	Sleep problems	Yes	168 (80.4)	41 (19.6)	0.948	1.02 (0.50; 2.10)
		No	48 (80.0)	12 (20.0)		1.00
	Urinary incontinence	Yes	50 (82.0)	11 (18.0)	0.770	1.12 (0.53; 2.33)
		No	167 (80.3)	41 (19.7)		1.00
	Digestive discomfort	Yes	74 (78.7)	20 (21.3)	0.619	0.85 (0.46; 1.59)
		No	143 (81.2)	33 (18.8)		1.00
	Sexually transmitted infections	Yes	19 (82.6)	4 (17.4)	0.704	1.24 (0.40; 3.84)
		No	168 (79.2)	44 (20.8)		1.00

**Table 4:** Odds ratio (OR) and 95% confidence intervals (95%CI) of the multiple regression model of variables associated with illicit substance use among homeless individuals. Jundiaí, 2023 (n=270)

Variables	Adjusted OR (95%CI)	p-value
**Education level**		
Complete high school or higher	4.16 (1.63; 10.62)	0.003
Complete elementary school	2.29 (0.90; 5.84)	0.082
Incomplete elementary school	1.00	
**Receiving any social assistance benefits**		
No	2.62 (1.28; 5.34)	0.008
Yes	1.00	
**Has suffered violence**		
Yes	2.05 (1.04; 4.04)	0.038
No	1.00	
**Smoker**		
Former smoker	7.64 (0.84; 69.47)	0.071
Yes	3.46 (1.60; 7.50)	0.002
No	1.00	
**Seeking health services when ill**		
No	1.54 (0.75; 3.13)	0.237
Yes	1.00	
**Time spent homeless**		
More than one year	2.08 (1.01; 4.28)	0.046
Up to one year	1.00	

## Discussion

The epidemiological profile revealed that the studied population was composed predominantly of men, single individuals, Black or Brown individuals, with a mean age of 39 years, low levels of education, living in extreme poverty, and experiencing homelessness for more than five years. This population showed high prevalence of violence, mental distress, and use of illicit substances, mainly crack, marijuana, and cocaine. Illicit substance use among the homeless population was associated with variables such as violence, tobacco use, time spent in street situations, access to social assistance benefits, and education. 

The invisibility and vulnerability of the homeless population are closely tied to social factors that influence health, worsening health risks ^18^. The study’s findings are consistent with other research on this population, revealing characteristics and behaviors that directly affect their health ^2^^,^^6^^,^^8^^,^^19^^,^^20^.

The most prevalent health conditions in the study population included mental distress, sleep problems, urinary incontinence, digestive discomfort, respiratory disease, dermatological disease, and hypertension. It is noteworthy that urinary incontinence was not previously identified in the literature as a health condition affecting homeless populations, possibly due to a lack of prior studies on this issue. Given the significant prevalence of this condition in the study territory, this may be considered an innovative aspect of this research. 

It was found that some homeless individuals do not practice personal hygiene. Studies indicate that significant tooth loss in this group is associated with age, poverty, and the absence of regular oral hygiene practices ^21^^,^^22^. Centro POP, located in downtown Jundiaí, is a facility where homeless individuals can attend to their personal hygiene needs. Nevertheless, due to heavy drug use and the central location of the Centro POP, which makes access difficult for those living in outlying areas, many homeless people fail to maintain this care.

Decree No. 11.908, dated February 6, 2024, established the Programa Brasil Saudável - Unir para Cuidar (Healthy Brazil Program - Unite to Care), aimed at eliminating diseases related to social factors. This program primarily focuses on individuals in contexts of high social vulnerability, such as those living on the streets. Its actions involve multiple governmental sectors, integrating health with housing, income generation, sanitation, education, and other public policies ^23^.

Society often stigmatizes homeless individuals, negatively labeling them and contributing to their marginalization ^24^^,^^25^. Each person carries a painful life history, and drug use may be a way of coping with social exclusion ^11^. The absence of state support exacerbates this vulnerability, creating a cycle that is difficult to break. The longer one remains on the streets, the greater the likelihood of turning to substances that harm health ^11^^,^^26^. Therefore, it is essential to adopt a humanized approach to this population and consider their life stories when formulating public policies ^27^.

The study shows that access to social assistance benefits, such as Bolsa Família, is inversely related to illicit substance use. Individuals who do not receive such benefits are more likely to consume these substances. Lack of information and bureaucratic barriers hinder access to social programs, especially among people experiencing homelessness, who are often excluded from the Brazilian National Health Registry ^28^. Thus, it is crucial to strengthen intersectoral actions to ensure fundamental rights and promote health, education, and social assistance services in areas with high levels of psychoactive substance use ^11^^,^^26^.

The association between illicit substance use and variables such as education, financial resources, family support, being a victim of violence, mental distress, sexually transmitted infections, and respiratory diseases has also been observed in the literature. This underscores the importance of understanding the complexity of this health behavior and the context of life on the streets to inform public health decision-making ^10^^,^^25^. Education, as a primary health determinant, was found to be associated with illicit substance use, with higher prevalence among individuals with at least a high school education. However, it is essential to critically analyze this association, as a higher level of education should not be considered a risk factor in itself. One hypothesis that may explain this relationship is that psychoactive substance use often acts as a trigger for individuals to leave their homes and sever family ties, leading to street situations, as evidenced by the present and related studies ^2^^,^^15^^,^^19^^,^^29^. 

Homeless individuals are exposed to various forms of violence-symbolic, physical, sexual, material, psychological, among others ^30^. The lack of adequate housing increases vulnerability to violence, which, in turn, contributes to the use of illicit substances ^2^^,^^5^^,^^30^. This study reveals that the context of rights violations and the lack of public policies create a cycle in which violence generates further violence. Moreover, a high rate of aggressive behavior was observed in this population, intensifying mental health issues and increasing vulnerability to psychoactive substance use, thus perpetuating this negative cycle ^5^^,^^29^.

In this study, smoking was associated with illicit substance use, a finding that aligns with previous research ^11^^,^^14^^,^^18^^,^^30^. Other studies have linked smoking to tuberculosis among homeless populations ^10^. The present study also identified a considerable incidence of respiratory conditions among the analyzed sample. Health education interventions are fundamental to raise awareness among vulnerable populations about the effects of smoking and to promote self-care strategies from a harm reduction perspective ^30^. 

Many challenges remain for the full implementation of health policies based on the principles of universality and comprehensiveness for social minorities, such as the population studied ^20^. Barriers faced by homeless individuals in accessing health services require strategic planning that prioritizes equity in care. Prejudice, the requirement of documentation, and a lack of welcoming attitudes hinder access to these services. Nonetheless, initiatives such as the Consultório na Rua (Street Clinic), when articulated with social assistance and psychosocial care centers, show potential to improve this access, even though the majority of participants in the study reported not experiencing difficulties ^2^^,^^18^^,^^24^. 

This study has limitations inherent to its cross-sectional design, which prevents the establishment of causality and the temporal sequence between illicit substance use and homelessness. Another limitation was the use of convenience sampling, which, although necessary for this research due to the characteristics of the study population and the difficulty of conducting random sampling, may limit the generalizability of the findings. Nonetheless, the study employed a sample defined by statistical criteria, with carefully selected variables, and is innovative in addressing the homeless population in Jundiaí, São Paulo. Scientific knowledge contributes to combating stigma and facilitates access and welcoming strategies that help reduce existing barriers ^6^^,^^11^^,^^20^. 

A relevant limitation of the study was that data collection was conducted in collaboration with the Street Clinic team, which may have introduced bias, as participants could have felt pressured to report access to health services, particularly those provided by the Street Clinic. Notably, 33.0% of respondents reported not using this service.

This study analyzed the sociodemographic and clinical-epidemiological profile of the homeless population and found a high prevalence of illness and illicit psychoactive substance use within this group. The factors associated with such use included sociodemographic variables, violence, and longer periods of living on the streets. These results underscore the importance of strengthening the health and intersectoral networks to ensure comprehensive care and guarantee social rights. 

## Data Availability

The database was made available on the OSF repository. The doi code is https://doi.org/10.17605/OSF.IO/M62PN
