# Supplementary material for: Epidemiological profile of the homeless population and factors associated with illicit psychoactive substance use: cross-sectional study, Jundiaí, 2023
Source: Epidemiol Serv Saude. 2026 Apr 22;35:e20240124. doi: 10.1590/S2237-96222026v35e20240124.en (PMC13686315; doi:10.1590/S2237-96222026v35e20240124.en)
Supplement: Translation

# Translation: Perfil epidemiológico da população em situação de rua e fatores associados ao uso de substâncias psicoativas ilícitas: estudo transversal, Jundiaí, 2023

**DOI:** 10.1590/S2237-96222026v35e20240124.pt

**Authors:** Gabriel Vinícius Reis de Queiroz, Tatiane Bahia do Vale Silva, Marília Jesus Batista

**Affiliations:** 1 Faculdade de Medicina de Jundiaí, Programa de Pós-Graduação em Ciências da Saúde, Jundiaí, SP, Brasil Faculdade de Medicina de Jundiaí Programa de Pós-Graduação em Ciências da Saúde Jundiaí SP Brasil; 2 Universidade do Estado do Pará, Departamento de Ciências do Movimento Humano, Belém, PA, Brasil Universidade do Estado do Pará Departamento de Ciências do Movimento Humano Belém PA Brasil; 3 aFaculdade de Medicina de Jundiaí, Departamento de Saúde Coletiva, Jundiaí, SP, Brasil Faculdade de Medicina de Jundiaí Departamento de Saúde Coletiva Jundiaí SP Brasil

**Keywords:** Pessoas Mal Alojadas, Vulnerabilidade Social, Epidemiologia, Drogas Ilícitas, Estudos Transversais.

## Abstract

**Objetivos:**

Descrever as variáveis sociodemográficas e clínico-epidemiológicas da população em situação de rua e avaliar os fatores associados ao uso de substâncias psicoativas ilícitas em Jundiaí, São Paulo, Brasil, em 2023.

**Métodos:**

Estudo transversal com uma população em situação de rua; o desfecho foi uso de substância, e as variáveis independentes foram agrupadas em exógenas, determinantes primários e intermediários em saúde, acesso a serviços, percepção de saúde e condições clínicas. Utilizou-se análise estatística descritiva e bivariada. Razão de chances (*odds ratio*, OR) com intervalos de confiança de 95% (IC95%) foram calculados por regressão logística binária múltipla.

**Resultados:**

Foram 270 participantes, sendo eles majoritariamente do sexo masculino (85,2%), com idade média de 39 anos, autodeclarados negros (72,6%), heterossexuais (88,5%), com ensino fundamental incompleto (54,8%), em situação de rua por cinco anos ou mais (45,6%) e 80,4% já usaram/fazem uso de substâncias psicoativas ilícitas. Foram mais prevalentes o sofrimento mental (83,0%), problemas no sono (77,4%), desconforto digestivo (34,8%) e doença respiratória (31,5%). O uso de substâncias ilícitas esteve associado a ter: escolaridade de ensino médio completo ou mais (OR 4,16; IC95% 1,63; 10,62); mais de um ano em situação de rua (OR 2,08; IC95% 1,01; 4,28); sofrido violência (OR 2,05; IC95% 1,04; 4,04); tabagismo (OR 3,46; IC95% 1,60; 7,50); e em não receber benefício socioassistencial (OR 2,62; IC95% 1,28; 5,34).

**Conclusão:**

Constataram-se comorbidades e alta prevalência de uso de substâncias psicoativas ilícitas associadas a determinantes socioeconômicos e comportamentais em saúde. Esses dados orientam a gestão pública em saúde na elaboração de estratégias para as necessidades específicas dessa população.

**Aspectos éticos**

Esta pesquisa respeitou os princípios éticos, obtendo os seguintes dados de aprovação:

Comitê de ética em pesquisa: Faculdade de Medicina de Jundiaí

Número do parecer: 4.915.622

Data de aprovação: 18/8/2021

Certificado de apresentação de apreciação ética: 48459121.4.0000.5412

Registro do consentimento livre e esclarecido: Obtido de todos os participantes antes da coleta.

## Introdução

A existência de pessoas em situação de rua é um fenômeno mundial e representa uma forte manifestação de desigualdade e de exclusão social provenientes da supervalorização do capital em desfavor dos direitos do ser humano ^1^. Esse grupo populacional apresenta fragilidade ou inexistência dos vínculos familiares, extrema pobreza e ausência de moradia convencional, utilizando espaços públicos e/ou abrigos como local de moradia ^2^. Segundo o Censo de 2021, realizado pela Prefeitura de São Paulo após o advento da pandemia, observou-se um crescimento de 31,0% na população em situação de rua no município de São Paulo, que aumentou de 24.344 pessoas em 2019 para 31.884 pessoas em 2021. Além disso, estima-se que 281.472 indivíduos vivam nessa condição em todo o Brasil, evidenciando um desafio significativo para as políticas sociais no país ^3^^,^^4^.

A problemática da vulnerabilidade, assim como as especificidades culturais e de estilo de vida da população em situação de rua, impacta diretamente os determinantes sociais da saúde ^5^^,^^6^. As pessoas em situação de rua são constantemente expostas a precárias condições de saúde, higiene, moradia, alimentação, exposição exacerbada ao calor e ao frio, além de a violência e/ou esforços físicos exaustivos para a sobrevivência, podendo apresentar o surgimento e/ou o aumento de problemas de saúde ^6^. A literatura aponta que as condições de saúde mais prevalentes nessa população são: tuberculose, pneumonia, sífilis, vírus da imunodeficiência humana, herpes genital, odontalgia, hipertensão arterial, sofrimento mental, parasitoses intestinais e consumo abusivo de substâncias psicoativas ^7^^,^^8^^,^^9^.

As razões que levam as pessoas a viverem nas ruas são variadas, com destaque para: violências domésticas, tragédias pessoais, conflitos familiares e uso de substâncias psicoativas ^10^. O consumo dessas substâncias é comum e serve como uma forma de evitar a dura realidade ^11^. Para entender esse uso, é importante enxergar essas pessoas como protagonistas de suas próprias histórias, levando em conta as suas vidas e seus contextos sociais, bem como a necessidade de uma rede de apoio e as influências culturais ^10^^,^^11^.

Esta pesquisa se mostra relevante ao abordar as condições de saúde da população em situação de rua, promovendo a visibilidade de um grupo social vulnerável. Tal iniciativa propicia a diminuição de desigualdades em saúde, além de combater estigmas e preconceitos persistentes. Portanto, torna-se imprescindível compreender as características dessa população, a fim de fornecer subsídios para a formulação de políticas públicas adequadas que reflitam as suas especificidades. Isso é crucial para o aprimoramento das estratégias de cuidado em saúde, fundamentadas na realidade que essas pessoas vivenciam e no entendimento das suas dinâmicas culturais, especialmente no que se diz respeito ao uso de substâncias psicoativas.

Assim sendo, o objetivo deste estudo é descrever as variáveis sociodemográficas e clínico-epidemiológicas da população em situação de rua e avaliar os fatores associados ao uso de substâncias psicoativas ilícitas em Jundiaí, São Paulo, Brasil, em 2023.

## Métodos

### Delineamento

Estudo epidemiológico transversal analítico reportado segundo as recomendações do Strengthening the Reporting of Observational Studies in Epidemiology (STROBE) para estudos observacionais.

### Contexto

A população do estudo foi composta por pessoas em situação de rua em Jundiaí, São Paulo, Brasil ^12^. Essa cidade está localizada a 57 km da capital do estado, São Paulo, e, segundo o Censo de 2022, realizado pelo Instituto Brasileiro de Geografia e Estatística, a população estimada foi de 443.221 habitantes, comum índice de desenvolvimento humano de 0,822 ^13^. 

### Coleta de dados

A coleta de dados foi realizada de agosto de 2022 a setembro de 2023, com o auxílio da equipe do Consultório na Rua. Esse serviço, criado em Jundiaí em abril de 2014, atende pessoas em vulnerabilidade social, principalmente aquelas que usam substâncias psicoativas e estão desconectadas das redes de saúde. Em Jundiaí, a equipe do Consultório na Rua conta com um coordenador, uma psicóloga, duas enfermeiras, dois redutores de danos, uma médica de família e uma médica psiquiatra.

A equipe do Consultório na Rua atua em áreas com grande concentração de pessoas em situação de rua, com foco sobre as periferias onde o uso de substâncias psicoativas é comum. Durante reuniões com pesquisadores, notou-se a importância da integração das diversas atividades. O contato com a população revelou uma resistência inicial por parte dela. Porém, com explicações sobre a pesquisa e a frequência das visitas, foi possível estabelecer um diálogo que permitiu a condução do estudo.

### Participantes

Foram incluídas neste estudo pessoas em situação de rua em Jundiaí, de ambos os sexos, com idade igual ou superior a 18 anos, sendo excluídas as pessoas em situação de trânsito (aquelas que estão indo para outro município e estão de passagem pelo município), aquelas que estiveram sob efeito de substâncias psicoativas (álcool e/ou drogas) no momento da coleta de dados e as pessoas com inabilidade de fala oral.

### Variáveis 

Adotou-se modelo teórico conceitual adaptado para a seleção das variáveis ^14^. Esse modelo, desenvolvido por Andersen e Davidson, visa identificar as variáveis essenciais para uma análise sistemática das diferenças na diversidade dos fatores que influenciam a saúde ^15^. Segundo esse modelo, as condições do ambiente externo, a estrutura de assistência à saúde e as características individuais da população exercem influências consideráveis nos comportamentos vinculados à saúde ^15^. As variáveis deste estudo estão agrupadas nos seguintes blocos, iniciando-se do mais distal para o mais proximal (Figura 1).

**Figura 1.** Modelo teórico conceitual para a seleção das variáveis

• Exógenas: idade (contínua), sexo (masculino; feminino) e raça/cor da pele (branco; preto; pardo; outras [indígena; amarela]).

• Determinantes primários em saúde: identidade de gênero (cis; trans), orientação sexual (heterossexual; homossexual; bissexual; pansexual), escolaridade (ensino médio completo ou mais; ensino fundamental completo; ensino fundamental incompleto), possuir profissão (sim; não), sustento financeiro (comércio/artesanato; construção civil; guardador de carro; coleta de materiais recicláveis; carregador de caminhão; faxina; jardinagem; profissional do sexo; outras) e receber algum benefício socioassistencial (sim; não).

• Determinantes intermediários em saúde: escovar os dentes (sim; não), tomar banho (sim; não), consumir bebidas alcoólicas (sim; não), ser tabagista (sim; não; ex-tabagista), usar preservativo (sim; não), quantidade de parceiros sexuais (nenhum; um ou mais), manifestação de dor no momento da entrevista (sim; não), qualidade do sono (muito bom/bom; razoável; ruim/muito ruim), se sofreu violência (sim; não) e se foi violento (sim; não).

• Acesso a serviços de saúde: consulta com profissionais da saúde (raramente/nunca; algumas vezes; frequentemente/sempre), se, quando doente, busca pelo serviço de saúde (sim; não), última vez que procurou o médico e/ou o dentista (há menos de seis meses; de seis meses a um ano; um a dois anos; três anos; acima de três anos; não sabe; nunca procurou), avaliação dos serviços de saúde que já utilizou (muito bom/bom; razoável; ruim/muito ruim), as dificuldades para acessar o serviço de saúde (forma de tratamento pelos funcionários; dificuldade de conseguir vaga; não conhece os serviços; falta de documentos; não teve dificuldade); Considerou-se como possibilidades para formas de tratamento pelos funcionários racismo, maus tratos violência, e desorganização.

• Percepção da saúde: classificação do estado atual de saúde (muito bom/bom; razoável; ruim/muito ruim), tempo em situação de rua (até um ano; mais de um ano), saúde após ficar em situação de rua (melhorou/melhorou muito; manteve-se; piorou/piorou muito) e contato com familiares (até uma semana; até um ano; perdeu contato/mais de cinco anos).

• Condições clínicas: hipertensão arterial (sim; não), doença respiratória (sim; não), doença dermatológica (sim; não), doença cardíaca (sim; não), doença neurológica (sim; não), diabetes (sim; não), doença infectocontagiosa (sim; não), dificuldade em controlar o uso do álcool (sim; não), sofrimento mental (sim; não), problemas do sono (sim; não), incontinência urinária (sim; não), desconforto digestivo (sim; não) e infecções sexualmente transmissíveis (sim; não), ambas autorrelatadas. 

A variável dependente (desfecho) deste estudo foi o consumo de substâncias ilícitas, sendo sim (usa e já usou) para o uso de substâncias ilícitas, e não (não usa e nunca usou) quando o participante declarou não usar essas substâncias. Com o intuito de descrever as características dessa população, perguntou-se sobre os tipos de substâncias ilícitas utilizadas, como crack, maconha, cocaína, ecstasy e drogas inaladas.

### Fontes de dados e mensuração

Elaborou-se um questionário baseado no instrumental de situação e na vulnerabilidade, o qual foi construído especificamente para a população em situação de rua e adaptado para este estudo, visando-se obter informações sobre aspectos sociodemográficos e condições de vida e saúde ^16^. O questionário conteve 58 questões no total, com perguntas abertas e fechadas. Primeiramente, o questionário foi validado pela equipe do Consultório na Rua e, posteriormente, foi realizado um estudo piloto no território com dez indivíduos. 

As entrevistas foram conduzidas pelo pesquisador e pelos membros da equipe do Consultório na Rua, estando todos previamente treinados. Inicialmente, abordou-se os participantes nos diversos territórios, conversando sobre o estudo e convidando-os para responder ao questionário. Após a confirmação de participação, aplicou-se o questionário. Seja na rua ou nos atendimentos dos equipamentos oportunos, tentou-se coletar os dados em espaços mais reservados.

### Tamanho do estudo

Dada à natureza transitória desse grupo populacional, escolheu-se a amostragem por conveniência, possibilitando abordar as pessoas em situação de rua em diferentes locais da cidade, enquanto a equipe do Consultório na Rua prestava-lhes serviço.

A amostragem foi efetuada utilizando-se a fórmula apropriada para populações finitas. Para o cálculo amostral, foi tomado como referência um estudo que investigou a saúde da população em situação de rua de Belo Horizonte, Minas Gerais, revelando que 47,35% dos entrevistados classificaram sua saúde como ruim ^17^. Adotou-se a significância de 5% e erro de 10%. O tamanho amostral final foi de 216 participantes. Estimando-se uma provável perda, adicionou-se a ela 20,00%, chegando-se ao total de 270 participantes.

### Métodos estatísticos

Os dados coletados foram submetidos e processados pelo programa Statistical Package for the Social Sciences (SPSS), versão 20.0. Inicialmente, realizaram-se as análises descritivas para alcançar a frequência e a média da idade. Posteriormente, realizaram-se análises bivariadas entre o desfecho, que foi o uso de substâncias ilícitas (sim e já usou, ou não usa e nunca usou), e as variáveis independentes do estudo por meio do teste qui-quadrado. 

Foi realizada análise de regressão logística binária univariada. As variáveis que apresentaram um p-valor<0,20 foram incluídas em modelos de regressão logística binária múltipla pelo método *step forward*. Foi testada a multicolinearidade. O exponencial de β neste estudo foi apresentado como razão de chances (*odds ratio*, OR) bruto para a análise univariada, e OR ajustado após a regressão logística. Realizou-se o cálculo do intervalo de confiança (IC95%). Adotou-se o nível de significância de 5%.

## Resultados

Das 270 pessoas em situação de rua com média da idade de 39 anos que compuseram a amostra desta pesquisa, 85,2% declararam ser do sexo masculino, 72,6% se declararam negros(as) (pretos e pardos), 88,5% heterossexuais, 54,8% possuem até o ensino fundamental incompleto, 28,5% utilizam a coleta de materiais recicláveis como principal meio de sustento financeiro, 47,8% não recebem nenhum tipo de benefício socioassistencial e 45,6% estão em situação de rua por mais de cinco anos. O uso de substâncias psicoativas (27,4%) e tragédias pessoais (21,1%) foram os motivos mais prevalentes para estar em situação de rua. Identificou-se que 80,4% dos participantes fazem uso ou já usaram algum tipo de substância ilícita, 48,1% utilizam crack, 41,5% fazem uso de maconha e 33,0% usam cocaína (Tabela 1).

Quanto às condições clínicas, 24,4% dos participantes apresentam hipertensão arterial, 31,5% declararam ter alguma doença respiratória, 33,0% possuem dificuldade em controlar o uso do álcool, 83,0% apresentam sofrimento mental, 77,4% problemas no sono, 22,6% incontinência urinária e 34,8% desconforto digestivo. Sobre o uso de serviços de saúde, 58,5% dos participantes avaliaram como bom os serviços e 67,0% relataram não ter nenhuma dificuldade para o acesso aos serviços de saúde. Entretanto, 22,6% deles mencionaram, como dificuldade no acesso, a forma de tratamento pelos funcionários, como racismo, maus tratos, violência e/ou desorganização (Tabela 2).

**Tabela 1:** Frequência (n) e porcentagem (%) das características sociodemográficas e comportamentos em saúde da população em situação de rua. Jundiaí, 2023 (n=270)

**Variáveis**			**n (%)**
**Características sociodemográficas**	Raça/cor da pele	Branco	69 (25,6)
		Preto	56 (20,7)
		Pardo	140 (51,9)
		Outras (indígena, amarela)	5 (1,8)
	Sexo	Masculino	230 (85,2)
		Feminino	40 (14,8)
	Identidade de gênero^a^	Cis	256 (94,8)
		Trans	13 (4,8)
	Orientação sexual	Heterossexual	239 (88,5)
		Homossexual	17 (6,3)
		Bissexual	13 (4,8)
		Pansexual	1 (0,4)
	Escolaridade	Ensino fundamental incompleto	148 (54,8)
		Ensino fundamental completo	52 (19,3)
		Ensino médio completo ou mais	70 (25,9)
	Possuir profissão	Sim	255 (94,4)
		Não	15 (5,6)
	Recebimento de benefício socioassistencial	Sim	141 (52,2)
		Não	129 (47,8)
	Sustento financeiro	Comércio/artesanato	27 (10,0)
		Construção civil	23 (8,5)
		Guardador de carro (flanelinha)	3 (1,1)
		Coleta de materiais recicláveis	77 (28,5)
		Carregador de caminhão (chapa)	3 (1,1)
		Faxina	4 (1,5)
		Jardinagem	4 (1,5)
		Profissional do sexo	11 (4,1)
		Outras	56 (20,7)
		Não trabalha	62 (23,0)
	Tempo em situação	Até um ano	78 (28,9)
	de rua	Um a quatro anos e 11 meses	68 (25,2)
		Mais de cinco anos	123 (45,6)
		Não sabe	1 (0,3)
**Comportamentos em saúde**	Classificação do estado de saúde atual^c^	Muito bom	6 (2,2)
		Bom	46 (17,0)
		Razoável	82 (30,4)
		Ruim	63 (23,3)
		Muito ruim	73 (27,0)
	Manifestação de dor no momento da entrevista	Sim	140 (51,9)
		Não	130 (48,1)
	Saúde após ficar em situação de rua	Melhorou muito	3 (1,1)
		Melhorou	8 (3,0)
		Manteve-se	50 (18,5)
		Piorou	57 (21,1)
		Piorou muito	152 (56,3)
**Comportamentos em saúde**	Motivo de estar em situação de rua^c^	Busca de trabalho	19 (7,0)
		Violência doméstica	47 (17,4)
		Abandono do grupo familiar	34 (12,6)
		Ameaças/conflitos	2 (0,7)
		Saída do sistema prisional	8 (3,0)
		Perda de tudo	16 (5,9)
		Tragédias pessoais	57 (21,1)
		Discriminação por orientação sexual	4 (1,5)
		Uso de substâncias psicoativas	74 (27,4)
		Outros	7 (2,6)
		Não sabe	2 (0,7)
	Escovar os dentes	Sim	225 (83,3)
		Não	45 (16,7)
	Tomar banho	Sim	259 (95,9)
		Não	11 (4,1)
	Ter sofrido violência	Sim	160 (59,3)
		Não	110 (40,7)
	Avaliação da qualidade do sono^c^	Muito bom	5 (1,9)
		Bom	38 (14,1)
		Razoável	27 (10,0)
		Ruim	25 (9,3)
		Muito ruim	175 (64,8)
	Consumo de bebidas alcoólicas^c^	Sim	183 (67,8)
		Não	86 (31,9)
		Não sabe	1 (0,4)
	Tabagista^c^	Sim	211 (78,1)
		Não	47 (17,4)
		Extabagista	12 (4,4)
	Uso de substâncias ilícitas	Sim	179 (66,3)
		Não	53 (19,6)
		Já usou	38 (14,1)
	Uso de crack	Sim	130 (48,1)
		Não	102 (37,8)
		Não se aplica^b^	38 (14,1)
	Uso de maconha	Sim	112 (41,5)
		Não	120 (44,4)
		Não se aplica^b^	38 (14,1)
	Uso de cocaína^c^	Sim	89 (33,0)
		Não	143 (53,0)
		Não se aplica^b^	38 (14,1)
	Uso de ecstasy^c^	Sim	15 (5,6)
		Não	217 (80,4)
		Não se aplica^b^	38 (14,1)
	Uso de drogas inaladas^c^	Sim	15 (5,6)
		Não	217 (80,4)
		Não se aplica^b^	38 (14,1)
	Uso de preservativo	Sim	155 (57,4)
		Não	112 (41,5)
		Não sabe	3 (1,1)

a Variável que não somou 100,0% devido à ausência de respostas (dados faltantes); ^b^O “Não se aplica” faz referência aos entrevistados que relataram já terem usado algum tipo de substância ilícita; ^c^Os percentuais foram arredondados para uma casa decimal. A soma pode não resultar exatamente em 100,0%.

**Tabela 2:** Frequência (n) e porcentagem (%) das características clínicas e uso de serviços em saúde pela população em situação de rua. Jundiaí, 2023 (n=270)

Variáveis			n (%)
Condições clínicas	Hipertensão arterial^a^	Sim	66 (24,4)
		Não	181 (67,0)
		Não sabe	23 (8,5)
	Doença cardíaca	Sim	17 (6,3)
		Não	241 (89,3)
		Não sabe	12 (4,4)
	Doença neurológica	Sim	21 (7,8)
		Não	237 (87,8)
		Não sabe	12 (4,4)
	Doença infecto contagiosa	Sim	20 (7,4)
		Não	239 (88,5)
		Não sab	11 (4,1)
	Doença respiratória	Sim	85 (31,5)
		Não	174 (64,4)
		Não sabe	11 (4,1)
	Doença dermatológica	Sim	32 (11,9)
		Não	236 (87,4)
		Não sabe	2 (0,7)
	Diabetes	Sim	20 (7,4)
		Não	227 (84,1)
		Não sabe	23 (8,5)
	Dificuldade em controlar o uso do álcool^a^	Sim	89 (33,0)
		Não	180 (66,7)
		Não sabe	1 (0,4)
	Sofrimento mental (depressão, ansiedade)	Sim	224 (83,0)
		Não	43 (15,9)
		Não sabe	3 (1,1)
	Problemas do sono	Sim	209 (77,4)
		Não	60 (22,2)
		Não sabe	1 (0,4)
	Incontinência urinária	Sim	61 (22,6)
		Não	208 (77,0)
		Não sabe	1 (0,4)
	Desconforto digestivo	Sim	94 (34,8)
		Não	176 (65,2)
	Infecções sexualmente transmissíveis (IST)	Sim	23 (8,5)
		Não	212 (78,5)
		Não sabe	35 (13,0)
Uso de serviços	Consulta com profissionais da saúde	Nunca	123 (45,6)
		Raramente	57 (21,1)
		Algumas vezes	56 (20,7)
		Frequentemente	23 (8,5)
		Sempre	11 (4,1)
	Busca pelo serviço de saúde	Sim	157 (58,1)
		Não	112 (41,5)
		Não sabe	1 (0,4)
	Última vez que procurou médico	Há menos de seis meses	158 (58,5)
		De seis meses a um ano	33 (12,2)
		Um a dois anos	23 (8,5)
		Três anos	6 (2,2)
		Acima de três anos	42 (15,6)
		Não sabe	6 (2,2)
		Nunca procurou	2 (0,7)
	Última vez que procurou dentista	Há menos de seis meses	48 (17,8)
		De seis meses a um ano	27 (10,0)
		Um a dois anos	33 (12,2)
		Três anos	2 (0,7)
		Acima de três anos	126 (46,7)
		Não sabe	5 (1,9)
		Nunca procurou	29 (10,7)
	Avaliação dos serviços de saúde que já utilizou	Muito bom	47 (17,4)
		Bom	158 (58,5)
		Razoável	39 (14,4)
		Ruim	11 (4,1)
		Muito ruim	15 (5,6)
	Dificuldades para acessar o serviço de saúde	Forma de tratamento pelos funcionários	61 (22,6)
		Dificuldade de conseguir vaga	11 (4,1)
		Não conhece os serviços	12 (4,4)
		Falta de documentos	5 (1,9)
		Não teve dificuldade	181 (67,0)

a Os percentuais foram arredondados para uma casa decimal. A soma pode não resultar exatamente em 100,0%.

A análise bivariada evidenciou associação entre o uso de substâncias ilícitas (desfecho) e: ter a escolaridade ensino médio completo ou mais (OR 3,22; IC95% 1,36; 7,62), não receber benefício socioassistencial (OR 2,51; IC95% 1,31; 4,78), ser tabagista (OR 4,14; IC95% 2,07; 8,26), ter sofrido violência (OR 2,23; IC95% 1,21; 4,10), ter promovido a violência (OR 2,56; IC95% 1,38; 4,75), estar a mais de um ano em situação de rua (OR 2,02; IC95% 1,08; 3,79), não ter hipertensão arterial (OR 2,10; IC95% 1,09; 4,03) nem diabetes (OR 4,40; IC95% 1,72; 11,25) (Tabela 3).

No modelo final de regressão, as variáveis que permaneceram significativas e associadas ao uso de substâncias ilícitas foram: nível de escolaridade ensino médio completo ou mais (OR 4,16; IC95% 1,63; 10,62), não receber benefício socioassistencial (OR 2,62; IC95% 1,28; 5,34), ter sofrido violência (OR 2,05; IC95% 1,04; 4,04), ser tabagista (OR 3,46; IC95% 1,60; 7,50) e estar vivendo em situação de rua há mais de um ano (OR 2,08; IC95% 1,01; 4,28) (Tabela 4).

**Tabela 3:** Razão de chances (*odds ratio*, OR) e intervalos de confiança de 95% (IC95%) do uso de substâncias ilícitas e sua associação com variáveis exógenas, determinantes primários e intermediários em saúde e condições clínicas entre pessoas em situação de rua. Jundiaí, 2023 (n=270)

Variáveis			Uso de substâncias ilícitas			
			Sim	Não	p-valor	OR (IC95%)
			n (%)	n (%)		
Variáveis	Sexo	Masculino	185 (80,4)	45 (19,6)	0,949	1,03 (0,44; 2,38)
exógenas		Feminino	32 (80,0)	8 (20,0)		1
	Raça/cor da pele	Branco	55 (79,7)	14 (20,3)	0,616	1
		Preto	47 (83,9)	9 (16,1)		1,33 (0,53; 3,35)
		Pardo	112 (80,0)	28 (20,0)		1,02 (0,50; 2,09)
		Outras	3 (60,0)	2 (40,0)		0,38 (0,06; 2,51)
**Determinantes primários em saúde**	Identidade de gênero	Cis	207 (80,9)	49 (19,1)	0,721	1,27 (0,34; 4,78)
		Trans	10 (76,9)	3 (23,1)		1
	Escolaridade	Ensino médio completo ou mais	63 (90,0)	7 (10,0)	0,008	3,22 (1,36; 7,62)
		Ensino fundamental completo	45 (86,5)	7 (13,5)		2,30 (0,96; 5,53)
		Ensino fundamental incompleto	109 (73,6)	39 (26,4)		1
	Recebimento de benefício socioassistencial	Sim	104 (73,8)	37 (26,2)	0,004	1
		Não	113 (87,6)	16 (12,4)		2,51 (1,31; 4,78)
**Comportamentos em saúde**	Escovar	Sim	181 (80,4)	44 (19,6)	0,945	1
	os dentes	Não	36 (80,0)	9 (20,0)		0,97 (0,44; 2,17)
	Tomar banho	Sim	210 (81,1)	49 (18,9)	0,154	1
		Não	7 (63,6)	4 (36,4)		0,41 (0,12; 1,45)
	Consumo de bebidas alcoólicas	Sim	152 (83,1)	31 (16,9)	0,147	1
		Não	65 (75,6)	21 (24,4)		0,63 (0,34; 1,18)
	Tabagista	Sim	179 (84,8)	32 (15,2)	<0,001	4,14 (2,07; 8,26)
		Não	27 (57,4)	20 (42,6)		1
		Ex-tabagista	11 (91,7)	1 (8,3)		8,15 (0,97; 68,38)
	Uso de preservativo	Sim	128 (82,6)	27 (17,4)	0,411	1
		Não	88 (78,6)	24 (21,4)		0,77 (0,42; 1,43)
	Quantidade de parceiros sexuais	Nenhum	139 (79,0)	37 (21,0)	0,43	1
		Um ou mais	78 (83,0)	16 (17,0)		1,30 (0,68; 2,48)
	Avaliação da qualidade do sono	Muito bom/bom	31 (72,1)	12 (27,9)	0,297	1
		Razoável	23 (85,2)	4 (14,8)		2,23 (0,64; 7,80)
		Ruim/muito ruim	163 (81,5)	37 (18,5)		1,71 (0,80; 3,63)
	Ter sofrido violência	Sim	137 (85,6)	23 (14,4)	0,009	2,23 (1,21; 4,10)
		Não	80 (72,7)	30 (27,3)		1
	Ter promovido violência	Sim	132 (86,8)	20 (13,2)	0,002	2,56 (1,38; 4,75)
		Não	85 (72,0)	33 (28,0)		1
**Acesso a serviços de saúde**	Consulta com profissionais da saúde	Raramente/nunca	151 (83,9)	29 (16,1)	0,063	0,74 (0,30; 1,86)
		Algumas vezes	39 (69,6)	17 (30,4)		0,44 (0,22; 0,82)
		Frequentemente/sempre	27 (79,4)	7 (20,6)		1
	Busca pelo serviço de saúde	Sim	120 (76,4)	37 (23,6)	0,059	1
		Não	96 (85,7)	16 (14,3)		1,85 (0,97; 3,53)
**Percepção da saúde**	Classificação do estado de saúde atual	Muito bom/bom	40 (76,9)	12 (23,1)	0,776	1
		Razoável	67 (81,7)	15 (18,3)		1,34 (0,57; 3,15)
		Ruim/muito ruim	110 (80,9)	26 (19,1)		1,27 (0,59; 2,75)
	Tempo em situação de rua	Até um ano	56 (71,8)	22 (28,2)	0,025	1
		Mais de um ano	160 (83,8)	31 (16,2)		2,02 (1,08; 3,79)
	Saúde após ficar em situação de rua	Melhorou/melhorou muito	8 (72,7)	3 (27,3)	0,782	1
		Manteve-se	41 (82,0)	9 (18,0)		1,71 (0,38; 7,74)
		Piorou/piorou muito	168 (80,4)	41 (19,6)		1,54 (0,39; 6,05)
	Contato com familiares	Contato em até uma semana	59 (81,9)	13 (18,1)	0,197	1,61 (0,77; 3,39)
		Contato em até um ano	41 (85,4)	7 (14,6)		2,08 (0,83; 5,19)
		Perdeu contato há mais de cinco anos	76 (73,8)	27 (26,2)		1
**Condições clínicas**	Hipertensão arterial	Sim	46 (69,7)	20 (30,3)	0,024	1
		Não	150 (82,9)	31 (17,1)		2,10 (1,09; 4,03)
	Doença respiratória	Sim	75 (88,2)	10 (11,8)	0,054	2,10 (0,99; 4,44)
		Não	136 (78,2)	38 (21,8)		1
	Doença dermatológica	Sim	26 (81,2)	6 (18,8)	0,877	1,08 (0,42; 2,77)
		Não	189 (80,1)	47 (19,9)		1
	Doença cardíaca	Sim	14 (82,4)	3 (17,6)	0,852	1,13 (0,31; 4,10)
		Não	194 (80,5)	47 (19,5)		1
	Doença neurológica	Sim	15 (71,4)	6 (28,6)	0,258	0,57 (0,21; 1,55)
		Não	193 (81,4)	44 (18,6)		1
	Diabetes	Sim	10 (50,0)	10 (50,0)	0,003	1
		Não	185 (81,5)	42 (18,5)		4,40 (1,72; 11,25)
	Doença infecto-contagiosa	Sim	17 (85,0)	3 (15,0)	0,774	1,35 (0,38; 4,80)
		Não	193 (80,8)	46 (19,2)		1
	Dificuldade em controlar o uso do álcool	Sim	71 (79,8)	18 (20,2)	0,794	0,92 (0,49; 1,74)
		Não	146 (81,1)	34 (18,9)		1
	Sofrimento mental	Sim	180 (80,4)	44 (19,6)	0,875	0,94 (0,41; 2,16)
		Não	35 (81,4)	8 (18,6)		1
	Problemas do sono	Sim	168 (80,4)	41 (19,6)	0,948	1,02 (0,50; 2,10)
		Não	48 (80,0)	12 (20,0)		1
	Incontinência urinária	Sim	50 (82,0)	11 (18,0)	0,77	1,12 (0,53; 2,33)
		Não	167 (80,3)	41 (19,7)		1
	Desconforto digestivo	Sim	74 (78,7)	20 (21,3)	0,619	0,85 (0,46; 1,59)
		Não	143 (81,2)	33 (18,8)		1
	Infecções sexualmente transmissíveis	Sim	19 (82,6)	4 (17,4)	0,704	1,24 (0,40; 3,84)
		Não	168 (79,2)	44 (20,8)		1

**Tabela 4:** Razão de chances (*odds ratio*, OR) e intervalos de confiança de 95% (IC95%) do modelo de regressão múltipla das variáveis associadas ao uso de substâncias ilícitas entre pessoas em situação de rua. Jundiaí, 2023 (n=270)

Variáveis	OR ajustado (IC95%)	p-valor
Escolaridade		
Ensino médio completo ou mais	4,16 (1,63; 10,62)	0,003
Ensino fundamental completo	2,29 (0,90; 5,84)	0,082
Ensino fundamental incompleto	1,00	
**Recebimento de benefício socioassistencial**		
Não	2,62 (1,28; 5,34)	0,008
Sim	1,00	
**Ter sofrido violência**		
Sim	2,05 (1,04; 4,04)	0,038
Não	1,00	
**Tabagista**		
Ex-tabagista	7,64 (0,84; 69,47)	0,071
Sim	3,46 (1,60; 7,50)	0,002
Não	1,00	
**Busca pelo serviço de saúde**		
Não	1,54 (0,75; 3,13)	0,237
Sim	1,00	
**Tempo em situação de rua**		
Mais de um ano	2,08 (1,01; 4,28)	0,046
Até um ano	1,00	

## Discussão

O perfil epidemiológico evidenciou que a população estudada é composta majoritariamente por: homens, solteiros, negros, com idade média de 39 anos, baixa escolaridade, em condição de extrema pobreza e em situação de rua por mais de cinco anos. Constatou-se, nessa população, alta prevalência de violência, sofrimento mental e uso de substâncias ilícitas, principalmente crack, maconha e cocaína. O uso de substâncias ilícitas pela população em situação de rua esteve associado às variáveis violência, tabagismo, tempo em situação de rua, recebimento de benefício socioassistencial e escolaridade. 

A invisibilidade e a vulnerabilidade da população em situação de rua estão ligadas aos fatores sociais que influenciam a saúde, agravando os riscos ^18^. Os dados do estudo corroboram outras pesquisas sobre essa população, mostrando características e comportamentos que afetam diretamente sua saúde ^2^^,^^6^^,^^8^^,^^19^^,^^20^.

As condições de saúde mais prevalentes na população estudada foram: sofrimento mental, problemas no sono, incontinência urinária, desconforto digestivo, doença respiratória, doença dermatológica e hipertensão arterial. Salienta-se que na literatura não foi identificada a incontinência urinária como uma condição de saúde das populações em situação de rua, talvez por não ser uma condição estudada anteriormente. Como se constatou prevalência significativa dessa afecção no território estudado, esse dado pode ser um aspecto inovador deste estudo. 

Identificou-se que algumas pessoas em situação de rua não praticam higiene pessoal. Estudos indicam que a perda significativa dos dentes nesse grupo está ligada à idade, à pobreza e à falta de cuidados regulares com a escovação dental ^21^^,^^22^. O Centro POP, situado no centro de Jundiaí, é onde as pessoas em situação de rua podem cuidar da higiene pessoal. No entanto, muitas delas, devido ao uso intenso de drogas e à distância da periferia, acabam não conseguindo manter esses cuidados.

O Decreto nº 11.908, de 6 de fevereiro de 2024, criou o Programa Brasil Saudável - Unir para Cuidar, com o objetivo de eliminar doenças ligadas a fatores sociais. Esse programa foca especialmente nas pessoas em contextos de alta vulnerabilidade social, como pessoas em situação de rua. As ações incluem diversos setores governamentais, integrando saúde com habitação, geração de renda, saneamento e educação, além de outras políticas públicas ^23^.

A sociedade muitas vezes estigmatiza as pessoas em situação de rua, rotulando-as de maneira negativa, o que contribui para sua marginalização ^24^^,^^25^. Cada indivíduo carrega consigo uma história de vida dolorosa, e o uso de drogas pode ser uma forma de lidar com a exclusão social ^11^. A falta de suporte estatal agrava essa vulnerabilidade, criando um ciclo difícil de romper. Quanto mais tempo alguém passa nas ruas, maior a probabilidade de recorrer a substâncias prejudiciais à saúde ^11^^,^^26^. Portanto, é fundamental humanizar a abordagem quanto a essa população e considerar as vidas dessas pessoas ao desenvolver políticas públicas ^27^.

A pesquisa revela que a concessão de benefícios socioassistenciais, como o Bolsa Família, está inversamente relacionada ao uso de substâncias ilícitas. Indivíduos não beneficiados apresentam maior propensão ao consumo de tais substâncias. A falta de informações e barreiras burocráticas dificultam o acesso a esses programas sociais, especialmente entre a população em situação de rua, que frequentemente permanece excluída do Cadastro Único de Saúde ^28^. Portanto, é imprescindível fortalecer a atuação intersetorial para assegurar direitos fundamentais, promovendo serviços de saúde, educação e assistência social em locais de consumo intenso de substâncias psicoativas ^11^^,^^26^.

A associação do uso de substâncias ilícitas com variáveis, como escolaridade, sustento financeiro, suporte familiar, vítima de violência, sofrimento mental, infecções sexualmente transmissíveis e doenças respiratórias, também tem sido observada no acervo literário, reforçando a pertinência em compreender a complexidade desse comportamento em saúde e o contexto do viver nas ruas para a tomada de decisão da gestão pública ^10^^,^^25^. A escolaridade, um determinante primário em saúde, foi associado ao uso de substâncias ilícitas, sendo que houve maior prevalência de uso em indivíduos com ensino médio completo ou mais. Contudo, é imprescindível analisar essa associação criticamente, visto que um nível educacional elevado não deve ser considerado um fator de risco. Uma hipótese que pode elucidar essa relação é que o uso de substâncias psicoativas frequentemente atua como um disparador para que os indivíduos abandonem seus lares e rompam vínculos familiares, resultando em situações de rua, conforme evidenciado neste estudo e em pesquisas correlatas ^2^^,^^15^^,^^19^^,^^29^.

As pessoas em situação de rua sofrem violência de diferentes modos, seja simbólica, física, sexual, patrimonial, psicológica, entre outras ^30^. A falta de moradia adequada expõe as pessoas a diversas formas de violência, o que, por sua vez, favorece o uso de substâncias ilícitas ^2^^,^^5^^,^^30^. Este estudo revela que o contexto de violação de direitos e a carência de políticas públicas criam um ciclo onde a violência gera mais violência. Além disso, foi observada uma alta taxa de comportamento agressivo entre essa população, intensificando problemas de saúde mental e aumentando a vulnerabilidade quanto ao uso de substâncias psicoativas, perpetuando ainda mais esse ciclo negativo ^5^^,^^29^.

Neste estudo, ser tabagista esteve associado ao uso de substâncias ilícitas, dado que corroborou outros estudos ^11^^,^^14^^,^^18^^,^^30^. Pesquisas anteriores relacionam o tabagismo com a tuberculose na população em situação de rua ^10^. Este estudo identificou uma considerável incidência de patologias respiratórias entre a amostra analisada. Intervenções educativas em saúde são fundamentais para conscientizar a população em situação de vulnerabilidade sobre os impactos do tabagismo e promover estratégias de autocuidado na perspectiva da redução de danos ^30^. 

Muitos são os desafios para a efetivação da política de saúde relacionada aos princípios da universalidade e da integralidade a minorias sociais, como a população estudada ^20^. As barreiras enfrentadas por indivíduos em situação de rua no acesso aos serviços de saúde demandam um planejamento estratégico que priorize a equidade no cuidado. O preconceito, a exigência de documentação e a falta de acolhimento dificultam essa assistência. No entanto, iniciativas como o Consultório na Rua, de forma articulada com a assistência social e os centros de atenção psicossocial, demonstram potencial para aprimorar esse acesso, embora a maioria da população da amostra indique não enfrentar dificuldades ^2^^,^^18^^,^^24^. 

Este estudo apresenta limitações atribuídas ao seu delineamento transversal, o que impede a determinação da causalidade e a sequência temporal do uso de substâncias ilícitas em relação à vivência em situação de rua. Outro aspecto limitador foi o modelo de amostragem por conveniência, embora necessário para a realização desta pesquisa, pela característica do grupo populacional estudado e pela dificuldade em realizar uma amostra aleatória a partir de um cadastro. Entretanto, o estudo examinou uma amostra definida por critérios estatísticos, focando na escolha cuidadosa das variáveis e sendo inovador ao abordar a população em situação de rua em Jundiaí, São Paulo. A produção científica ajuda a combater preconceitos e colabora no acesso e no acolhimento, que minimizam as barreiras existentes ^6^^,^^11^^,^^20^. 

Uma limitação relevante da pesquisa foi a coleta de dados em colaboração com o Consultório na Rua, o que pode ter introduzido um viés, uma vez que os participantes podem ter se sentido pressionados a relatar acesso aos serviços de saúde, especialmente os oferecidos pelo Consultório na Rua. Notavelmente, 33,0% dos entrevistados afirmaram não utilizar esse serviço.

Este estudo analisou o perfil sociodemográfico e clínico-epidemiológico da população em situação de rua e observou o adoecimento desse grupo com grande prevalência de uso de substâncias psicoativas ilícitas. Os fatores associados a esse uso foram sociodemográficos, violência e maior tempo em situação de rua. Esses resultados direcionam para a importância de fortalecer a rede de saúde e intersetorial, visando assegurar a integralidade do cuidado e a garantia de direitos sociais. 

## Data Availability

O banco de dados foi disponibilizado no repositório OSF com doi: https://doi.org/10.17605/OSF.IO/M62PN.
